# Commentaries on Some of the More Important of the Diseases of Females

**Published:** 1827-10

**Authors:** 


*Commentaries on some of the more important of the Diseases of Females. In Three Parts.* By Marshall Hall, M.D. F.R.S. E. Octavo, pp. 376, with Eight coloured Plates. Longman and Co's. 1827.

Dr. Hall's name has been so long familiar to our readers, that it is unnecessary to recal to their recollection the several works which he has published—and which have uniformly characterised him as a most acute as well as talented observer of the phenomena of diseases. It is now nearly ten years since his Essay on the "Mimoses, or Disorders of the Digestive Organs," attracted considerable attention, and stamped the author as a physician far beyond the general run of routine practitioners, who too often take for granted what has been first impressed on their minds by their teachers, and dislike the trouble of afterwards observing, comparing, investigating, and thinking for themselves. The only fault, indeed, which has been objected to Dr. Hall, is that of being too minute in his distinctions—and if this was a *fault*, it was founded on his being too minute and accurate in his observance of the various shades and differences of diseases—a supererogation that will be readily pardoned!

The present work is not original—but a consolidation and emendation of the author's former publications, in which he has entered more fully into the investigation of those diseases which formed the subjects of his essays and papers, "in the conviction that a renewed inquiry into their diagnosis or essential differences under a similarity of appearances, and into their nature, pathology, and treatment, will lead to many important and beneficial results."—*Pref.* The author modestly considers this inquiry "as only just begun, and as still affording great scope for further investigation."

These commentaries are divided into three parts—the first embracing those morbid affections incident to female youth—the second comprehending the disease which supervenes during the puerperal state—and the third treating of those affections incident to the middle and later periods of female life. To each of these three parts we shall dedicate an analytical article, passing lightly, of course, over those subjects which have been already reviewed in this series of our Journal.

### I. *Disorders incident to Female Youth.*

Dr. Hall thinks that the peculiarity of the female constitution which principally modifies the disorders of the sex, depends chiefly on "the greater development of the capillary vessels, and the greater susceptibility of the nervous system, than are observed in the male sex." Hence results a greater tendency to those affections attended with pallor, œdema, and even hæmorrhagic effusions. The female is evidently of more sensitive nerve than the male, and is more subject to painful affections of the head, heart, side, and other parts of the body. The growth of the body influences the general health, and the general health influences, in turn, the growth of the body. A

too rapid growth involves debility—while debility often impedes the growth, and interrupts the building up and proportioning of the spine, the pelvis, &c.

The epoch of primary menstruation, like the growth of the body, has a considerable influence on the health, and *vice versa.* So, also, derangements in the return and flow of the catamenia, while they are almost always the effect of some disorder of the general health, become, in turn, a source of constitutional disorder. The state of the uterus and its secretion influences other parts of the body, and especially the mamma, which usually swells and becomes tender at the approach of puberty and the menses.

But there are other states common to the sex, which greatly modify their health; and among these, the confined and loaded condition of the bowels deserves great consideration, together with the injurious effects of sedentary habits. The want of sufficient corporeal exercise draws with it a defective peristaltic action of the intestines, and consequently a remora and vitiation of the fæcal remains of the food. To this may be added the inattention to the calls of nature. Does the ampler size of the pelvic cavity tend to the accumulations in the bowels of females? Dr. H. seems to speak in the affirmative. This remora, he observes, deranges the whole of the chylopoietic functions.

"And, first, the state and functions of the parts within the mouth become obviously disordered; the secretions are morbid, the tongue loaded and swollen, the gums and internal parts of the cheeks red and tumid, the teeth decayed, the breath tainted, and the saliva sometimes profuse and offensive.

"The complexion and general surface of the body then become morbidly affected, and there are pallor, icterode, and other hues, morbid states of each of the textures composing the skin, and frequently œdema. These conditions of the complexion, and of the general surface, vary both in their seat and appearance with the kind and state of the original disorder, and with the state of the tongue and internal mouth. With each of these associated appearances there is a peculiar condition both of the functions of the intestinal canal, and of that of the uterus; and all these affections are variously and characteristically modified by the duration of the malady."

The organs of digestion are disordered—nutrition is impeded—strength is reduced—and the liver (our author affirms) is frequently enlarged. Dr. Hall, however, cautions the medical practitioner against considering every icterode hue in the complexion or general surface as indicating disease or even disorder of this organ. "This state of the cutaneous surface is frequently the effect of a loaded condition and impaired function of the alimentary canal; and it is, in various instances, an affection of each of the cutaneous textures, or of the cutaneous circulation, altogether independent of any tinge of bile."

No organ, Dr. H. observes, is more influenced by the state of the intestinal canal than the uterus. With the changes observed in the state of the tongue, complexion, and cutaneous surface, "precisely proportionate changes take place (Dr. H. avers) in the state of the uterine discharges." The state of these discharges may, he says, in many instances, be ascertained by that of the tongue and complexion.

Dr. H. again adverts to the baleful effects of sedentary habits, and the almost total neglect of regular and active exercise in the open air. The occasional and formal promenade of a string of boarding-school misses in the train of their governess, and that only on a fine day, is a mere mockery of that vigorous muscular exercise which nature prompts and craves for in early life.

### II. *Disorder of the general Health—Acute Form.*

The general character and symptoms of this disorder are (Dr. H. thinks) very distinct; but its complications are very various, and sometimes predominate over the other symptoms, so as to resemble certain topical affections, and thus lead the practitioner astray.

"This affection, even in its more acute form, comes on insidiously, and the patient becomes gradually and insensibly incapacitated for exertion, either of mind or body. This state of unconscious disorder perhaps endures for many months, before it attracts the serious attention of the patient or her friends; and when a medical opinion is taken, it is usually sufficiently characterized by a general feeling of weakness, with tremor, head-ach, or vertigo, fluttering, faintishness, tendency to perspiration on the least exertion or surprise, susceptibility to hurry and agitation, weariness, aching, and loss of flesh: and with these symptoms there are peculiar states of the countenance, of the tongue and internal mouth, of the general surface, and of the evacuations, which I now proceed to describe. I would first observe, however, that although the accession of this morbid affection is usually slow and insidious, it is occasionally more rapid, being induced by the occurrence of some other indisposition, or of a fall or other accident.

"Soon after the commencement of this disorder, the countenance is observed to have become rather pale and thin; the lips are pale, and, with the chin, are frequently observed to be tremulous on speaking; the surface of the face is frequently affected with an appearance of oily and clammy perspiration, especially about the nose; and there is usually a degree of sallowness and darkness of the complexion in general, but principally about the eyes and mouth. The face is sometimes rather bloated, and the skin coarsish, at first; but afterwards there is some degree of emaciation.

"The tongue is almost invariably much loaded; sometimes, however, only slightly, whilst its edges are clean and red. In other severer cases, a load has been formed over the tongue, and has peeled off all at once, or in patches, leaving the suface of the tongue morbidly red, smooth, and tender. But generally the tongue in the acute form of disorder of the general health, is loaded, swollen, and œdematous, marked by pressure against the contiguous teeth, and formed, more or less, into sulci or plaits, and presents upon its upper surface numerous enlarged papillæ; the gums are also swollen, and sometimes red, at others palish, and they occasionally bleed; the inside of the cheeks are, like the tongue, frequently impressed by pressure against the teeth. This state of the tongue and of the gums is accurately represented in Plate I. figures 1, 2, and 3. To observe the sulci in its surface, it is sometimes necessary to distend the tongue by the pressure of two fingers, separating them in a lateral direction. The indentations in the edges of the tongue are most obvious early in the morning; but they are, as well as those in the inside of the cheeks, always obvious enough on a careful inspection. With this state of the surface of the tongue, gums, and cheeks, there is frequently a slight degree of morbid redness, and, perhaps, of tumidity about the tonsils and soft palate; the teeth, and the mouth in general, are foul; the saliva viscid, especially in the throat; and the breath tainted and fœtid; and I have occasionally known a degree of bleeding to take place, not from the gums alone, but from the posterior parts of the mouth; and this has chiefly occurred during the night, so that the patient has been awoke, and has probably been greatly alarmed, by finding a certain quantity of blood in her mouth.

"The tendency to perspiration is observed on the slightest surprise or exertion, and occasionally, though not very often, in the night, or early in the morning; the skin is, in general, cool, rather moist, and clammy; the hands and feet are apt to be cold, the fingers rather livid, and the nails frequently assume a lilac hue: this state of the hands accords a little with the representation given in Plate VIII. figure 1, but not entirely, the tips of the nails becoming opaque only in some protracted cases of disorder of the general health.

"The patient is frequently affected with nervous tremor, observed in a quivering of the lip or dimpling of the chin on speaking, or when at all agitated. There is also tremor on holding out the hand, or on carrying a cup of tea, for instance, to the mouth, on attempting to stand erect or walk, or on being fatigued or hurried. The tremor, in some cases, has formed the most remarkable feature of this affection; in others, it has been far less observed; but it is rarely, if ever, entirely absent. The patient feels unaccountably feeble and weary, and suffers from a sense of aching after slight exertion.

"There is an early, but very slow and gradual emaciation; and it is most interesting to remark, by weighing the patient, first, the still continued progress of the loss of flesh for a time; and then the cessation, and, lastly, the restoration from this fearful morbid process, on the institution of a correct mode of treatment.

"The patient experiences head-ach or vertigo, and much nervousness and susceptibility to hurry and agitation. There is sometimes heaviness for sleep; sometimes great wakefulness and restlessness; sometimes incubus; and, sometimes, though rarely, delirium; occasionally, there is loss of memory and absence of mind. Indeed the mental, sentient, and nervous powers appear all to be much under the influence of this affection of the general health.

"The patient is liable to experience faintishness in the upright position, if it be sustained for a little time. There is almost universally a peculiar sense of fluttering about the heart and pit of the stomach. The pulse is frequently nearly natural, but it is sometimes rather frequent, and it is easily accelerated. It is also apt to become irregular and intermittent." 21.

The state of the appetite accompanying the disordered condition of mouth already described, is various—sometimes impaired, sometimes unnaturally craving, without the power to take food. The digestion is apparently good in some—in others, it is attended with distention, flatus, eructation, pyrosis, and even vomiting. The bowels are at first constipated—afterwards relaxed and confined alternately—the evacuations being unnatural in both states, especially in the relaxed condition. Pain in some part of the colon or rectum is not uncommon. The urine is occasionally loaded at the beginning; but afterwards pale. The uterine discharges would probably be found to correspond with the others, if accurately observed—but this can seldom be done. Dr. H. adverts to the *constancy* of the morbid appearances in the countenance, mouth, and tongue—as contrasted with the variability of the other morbid phenomena. Hence, it is on the former chiefly that the accuracy of the diagnosis must rest.

After these observations on the general character of the complications, our author, with his wonted minuteness, proceeds to describe each of these complications separately, with the view of pointing out the diagnosis between them and idiopathic diseases of the same organs. He regrets that the diagnosis is frequently to be founded on the general symptoms, since an idiopathic and organic affection of some part may coexist with disorder of the general health, and, consequently, what was a mere functional complication in the beginning, may become organic in the sequel. He properly observes that it is always a suspicious omen when relief does not follow the due administration of remedies for the primary disorder, especially when the head is affected.

"The most usual affections of the head in this disorder, are pain and vertigo. If there be the symptoms of acute disorder of the general health already described, and if the head-ach or the vertigo has become habitual, or has long subsisted in a varying character, and especially if the usual remedies for fulness of the blood-vessels of the head have been tried without relief, or with transitory relief only, it may be presumed that the affection of the head is symptomatic only, and that it will yield to the plan of effectual but cordial purgatives, to be described hereafter. This opinion will be further substantiated, if the head-ach or vertigo be conjoined with sickness, faintishness, or cold perspiration, or if there be paleness of the countenance, and some degree of loss of flesh; and still further, if relief be obtained by the administration of warm aperients and a regulated plan of diet." 27.

During the whole course of this disorder, the patient is peculiarly liable to fluttering, irregular action, or violent palpitation of the heart, or even syncope. If there be organic disease of this organ, exertion will always exasperate it—if it be only functional, then, in the intervals of attacks, exercise can be borne without inconvenience. Hæmatemesis and melena are not uncommon complications of this form of the nameless or Proteiform malady, formerly designated by the term MIMOSIS, or imitator, by our talented observer, which term we find he has now dropped. The hæmatemesis is, of course, obvious enough; but the melena may exist without detection. Dr. H. thinks that it frequently occurs unknown and unsuspected by the patient. Jaundice is another complication, together with pains in various parts of the body, giving suspicion of gall-stones, inflammation of the pleura, liver, spleen, kidney, or other part of the body. "There is sometimes a sensible hardness, or tumour, which appears to consist in a loaded state of the intestines." Sometimes there is pain so severe as to resemble TIC DOULOUREUX—sometimes spasmodic or convulsive affections—sometimes such loss of muscular power, especially in the lower extremities, as to resemble paralysis. "Each of these cases is to be distinguished by a reference to the constitutional symptoms."

### TREATMENT.

The first object is to evacuate and regulate the bowels.

"It would be difficult, however, to determine whether more benefit has accrued from the use, or harm from the abuse, of purgative medicines in the present day. It does not appear to have been observed, that if these medicines be given unduly, they induce or keep up, in many instances, the very disorders they were intended to remove. This is true, not only in regard to the stomach and bowels themselves, but also in regard to some of those organs which are so apt to be affected symptomatically. In fact, if the due limits in giving purgative medicines be exceeded, a state of irritation and distention is maintained in the alimentary canal, and of exhaustion and nervousness in the general system, more distressing than the original disorder." 30.

This is a doctrine which we have been preaching for some years—and the longer we live, the more the evidence of its truth accumulates.

Purgative medicines should, therefore, be given (where their employment is necessary for a long time) in such manner and combination as not to produce the effect of keeping up irritation in the line of the alimentary tube. "This is to be done (says Dr. H.) by conjoining some cordial with the purgative, and by guarding against too considerable or too repeated doses of it; and by attention, at the same time, to a mild and nutritious diet."

After a free purgation, therefore, at the beginning, the object is to induce one full and consistent evacuation daily, avoiding, as much as possible, all teazing and irritating operation of medicine.

"The decoction of aloës, the infusion of rhubarb, and of senna, the tinctures of aloës, rhubarb, and senna, the vinum aloës, the Rochelle and Epsom salts, and manna, and aloës and rhubarb, variously formed into pills, are the remedies which I have thought most suited to effect the object which I have described." 31.

In cases where aperient medicine has proved irritating, five or six drops of laudanum, with twenty of liq. ammoniæ, added to the opening draught, have proved useful. Mercurials, in this class of complaints, are not often employed by Dr. Hall, except where the alvine evacuations do not resume their healthy aspect by ordinary means. Neither is our author very friendly to cordials. "They only excite feverishness; and, when the bowels are brought to a natural state, and when irritation and exhaustion are obviated, medicine has done its office." The rest is to be accomplished by diet, exercise, air, sponging the body, &c. On the subjects of diet, clothing, &c. our author is judicious, and his observations are entitled to respectful attention.

Before quitting the subject of the treatment in acute disorder of the general health, Dr. H. makes a few observations on the remedies for its various complications. On these we shall not dwell, as it is obvious that the primary object is to treat the general disorder, directing attention to the local or symptomatic affections, only when they become prominent or distressing. Thus, affections of the head must be met by cupping or leeching till the brain is relieved, if there be evidence of vascular action there; but it will not seldom require all the penetration of the practitioner to discriminate between head-ache from gastric and intestinal irritation, and that which results from actual fulness of blood, or inflammation of the membranes. Dr. H. remarks that, "in all those cases attended by hæmorrhage, he has been accustomed to prescribe the pilula hydrargyri." We should like to know his reasons for this. Our author is very friendly to the use of injections of warm water, in loaded, costive, or disordered states of the bowels, as a remedy that "may be beneficially substituted for much of the purgative medicine ordinarily prescribed, especially in females." It certainly is a very useful auxiliary; but we have reason to know that it will not often do without the aid of medicines which act on the upper bowels, and bring disordered secretions within the reach of the injection.

### CHAP. III. *Protracted General Disorder.*

The transitions from the more acute to the more protracted forms of disorder of the general health, are quite imperceptible. The affection may, Dr. H. observes, be considered acute perhaps during the first year of its existence. But it has generally subsisted some years before it assumes the characteristic form about to be described. In some cases there would not appear to have been any preceding acute form of the disorder, the malady having stolen imperceptibly on the patient. In this form, the states of the complexion, of the tongue and internal mouth, of the general surface, and of the bowels, are even more strongly characterised than in the more acute form of the disorder. But "the complications," observes Dr. H. "are usually of a totally different character."

"The countenance, in the protracted form of disorder of the general health, has gradually assumed a state of permanent paleness and sallowness, which are, however, by no means very considerable in all cases; the prolabia have lost the hue of health; and together with a diffused sallowness, a more morbid discolouration is usually observed occupying the eyelids, and encircling the mouth. The surface of the face is not affected with perspiration, as in the acute form of this disorder, nor is there the same degree of nervous tremor.

"The state of the tongue is most remarkable. It has, in the first place, generally become gradually clean and free from load; together with the whole internal mouth, it has lost its clamminess, its mucous covering, and its halituous appearance; and the secretions of the mouth and the breath are less offensive, and I have known them to acquire the peculiar odour of new milk. The morbid character of the tongue is evidently not of recent formation; it has no longer those acute impressions from its pressure against the teeth, observed in the acute form of this disorder; the indentations are still very marked, however, but their edges are rounded off; the sulci on the surface of the tongue are, in many instances, still more marked even, but they also have assumed a different character, evidently the impress of long duration; the papillæ are frequently still more enlarged, being much elongated, in some cases, and expanded laterally in others." 43.

These appearances are admirably portrayed in the second plate. Dr. Hall affirms that, "from the peculiar modification of morbid appearances in the tongue, we are also enabled to judge distinctly of the *actual state* as well as of the stage of the disorder." Our author says we can, from the state of the tongue, "form an accurate *conjecture* relative to the length of duration of this morbid affection." We hope Dr. Hall has not over-rated the diagnostic value of lingual phenomena. We have, all our lives, paid much attention to the appearances of this organ in diseases; but we confess we have not arrived at any thing like the accuracy of diagnosis which Dr. Hall has acquired.

Dr. Hall informs us that the *lobulated* appearance of the tongue has been often found by him to accompany simple enlargement of the liver, "of which disease it is therefore a symptom." The general surface, in this state of disorder, is very various—generally sallow and dry, or at least not perspirable. The nails also undergo some remarkable changes. "They first become dry, then brittle, and then begin to split at their points, until at length the patient cannot take a pin out of her dress without breaking them; afterwards they sink in irregularly in their centre, turning upwards at their points, which are variously cracked and split."

"In connexion with the general surface, it is important to remark, that the patient affected with protracted disorder of the general health is peculiarly subjected to several morbid states of the integuments, and of those internal surfaces which are immediately continued from the skin, such as, furunculus, paronychia, hordeola, erysipelas, especially of the nose, erythema nodosum, urticaria chronica, lichen, a harsh and cracked state of the skin surrounding the prolabia, purpura, inflammation, ulcerations or pustules of the conjunctiva, decay of the teeth, a morbid state of the gums and internal mouth, aphthæ of the tongue and inside of the lips or cheeks, chronic sore throat, deep ulcerations of the tongue, &c. and the occurrence of these local affections should always lead us to enquire into the state of the general health.

"There are, in the protracted form of this disorder, less tremor, debility, loss of flesh, and tendency to faintishness, and perspiration, than in the acute, although they are by no means entirely absent. The patient is still incapable of exertion, apt to perspire from effort or agitation, and subject to symptoms of affection of the head, heart, chest, and of the digestion; only usually in a slighter degree than is observed in the acuter form of this affection.

"The bowels, especially, are in an extremely deranged state, either constipated, or with scanty fluid, and fetid evacuations, and, more frequently than is suspected, mixed with blood.

"The uterine discharges become retarded in their returns, of short duration in their flow, discoloured, or pale, and scanty, frequently attended by much pain, and often succeeded by leucorrhœa.

"The appearance of the urine is, like all the other symptoms, very variable; sometimes the patient becomes subject to gravel.

"There is sometimes a degree of œdema, and sometimes a state of cachexia." 48.

The treatment is the same as in the acute form of disorder, excepting that more caution must be used in the exhibition of the different remedies—especially of purgatives.

"In addition to the former plan, there are questions, in the present case, respecting several other remedies; as the sarsaparilla, the sulphate of quinine, and the sulphate of iron. The two first, I am of opinion, may be safely given, and will be found of considerable advantage. The sulphate of iron requires rather more precaution in its administration, but is, I believe, a more efficacious remedy, when suitably given, than either of the former. In order that the sulphate of iron may be prescribed with advantage, the bowels must have been first freely evacuated, and then properly regulated for some time; the tongue must be clean, and the prolabium and countenance in general pale. In such a case, one grain of sulphate of iron, with some aromatic, may be given three times a day; and I think those times should be chosen when the stomach is not empty. I have sometimes even thought it advantageous to give this medicine during meals.

"The sarsaparilla, sulphate of quinine, and sulphate of iron, may also be advantageously given together." 50.

### CHAP. IV.—*Chlorosis*.

Dr. Hall is of opinion that this curious affection arises from the same causes of disorder of the general health as have already been detailed, and that the causes "of a sexual character," which have been assigned by authors, have rarely any thing to do with the complaint. It steals on in a very insidious manner, but has, according to our author, three pretty distinct stages. "It may be characterized in general as uniting a morbid paleness of the complexion, tongue, and general surface, with recurrent pain of the head, or of the side, palpitation, fluttering, and nervousness, and frequently attacks of hysteria—with some tendency to loss of flesh and to œdema." The various features of this disease, as portrayed in the general surface, the tongue, the countenance, the different functions, and the several secretions and excretions, are delineated and described by our author with more than German minuteness, and we must say with fidelity. For these descriptions we must refer to the volume itself, from page 51 to page 59.

Dr. Hall despairs of giving any thing accurate or specific in regard to the pathology of this and other forms of disorder of the general health. "There appears to me not to be a system, an organ, a texture, or even a fluid in the animal economy, which does not suffer in different instances of this multiform disorder."* A page farther on, however, Dr. Hall gives it as his opinion, that "the first cause is in the state of the bowels"—that "a concurrent cause is the peculiarity of constitution already described (humoral pathology)—and that an exciting cause is the inactive and sedentary mode of life usually obtaining in female youth." The stomach, Dr. H. thinks, suffers from its contiguity with the intestines; but really we see no reason why the stomach should not suffer from the same general causes that act on other parts of the system, including the intestines, and that as soon as any other organ or structure in the body. We agree with our author, however, that it is in the digestive organs the first and principal manifestation of the Proteian malady (of which chlorosis is merely one of the numerous shapes) is to be found. This *effect* of numerous causes, moral and physical, becomes, in turn, the *cause* of the numerous other phenomena presented in various parts of the body. That the state of the circulating fluids is deteriorated in this complaint there can be little doubt; and this deterioration of the blood may well become a cause of impaired vital energy in the heart, brain, and other organs. The painful affections of parts, in chlorosis, have sometimes been mistaken for organic disease; and the means employed as remedies have then increased the malady; but a careful examination of the constitution will readily detect the nature of the complaint.

"The treatment of this form of disorder of the general health must

* "There is in chlorosis a remarkable state of the capillary system of circulation, both of the vessels and of the fluids; it is this which gives origin to the exsanguious appearance of the countenance, prolabia, tongue, gums, and general surface; to the tendency to œdema; and to different species of hæmorrhages, especially those of the mucous and cutaneous surfaces, as epistaxis, melæna, hæmatemesis, and even purpura; and it is from this circumstance that the catamenia become almost colourless and aqueous. I have observed the blood which has flowed from the nose scarcely to tinge the sheets, and that taken from the arm, to resolve itself almost entirely into serum, with scarcely any crassamentum. This disorder affords, therefore, one of the most unequivocal examples of humoral pathology." 61.

be begun by a due evacuation of the bowels; but the use of mercurials, and of active purgatives in general, requires still greater precaution, and the addition of mild cordial remedies is still more necessary, even than in the forms of disorder described in the two preceding chapters.

"Of the class of aperient remedies, aloës and rhubarb appear to me to be best adapted to the cure of chlorosis; the first of these may be given in the form of the decoction, the wine, the simple and compound tinctures, the latter in those of the infusion and the tinctures, and these may also be variously combined together, and, if quite necessary, with manna, and the Rochelle salt.

"When the bowels have, by these means, been fully but gently regulated for some time, different preparations of iron, but especially the sulphate, become specific in this disorder,—gradually restoring the complexion, the general surface, and the uterine discharges, to their healthy state. The condition of the complexion, and of the catamenia, constitutes the true indication for the employment of these remedies, and of their beneficial effects when these begin to be displayed. I do not mean to confine the use of chalybeates to the cases in which the prolabia are exsanguious and the catamenia are pale, but I can affirm that their efficacy is most unequivocal in these cases.

"The painful affections of the head, of the sides, and of the abdomen, which are so apt to occur in complications of this morbid affection, are generally soon removed by an attention to the original disorder; but, in the mean time, they admit of being much relieved by the application of a spirituous lotion to the former part, or of a liniment composed of the soap liniment, the sal volatile, and the liquor ammoniæ, to the latter, or, if necessary, by a blister. If any further remedy be required, cupping may be tried; but it is extremely important, in chlorosis, to avoid taking blood as much as possible." 67.

Dr. H. remarks, in conclusion, that there is a peculiar species of dropsy, of which he has not seen many instances, but which appears to be allied to this disorder. It is attended by all the phenomena of chlorosis, "and it would probably yield to the remedies of this morbid affection."

Chap. V.—This chapter is devoted to certain forms of disordered health, attended by other changes in the complexion, besides those already noticed. These are chiefly the following, viz. first, the icterode, or yellowish—secondly, the light lead hue—and, thirdly, the ring of tumid darkness occupying the eye-lids. Dr. H. avers, that all these morbid states of the complexion are "dissimilar from that observed in chlorosis, and from each other." "Each denotes a distinct form of disorder, having distinct characters and tendencies, being associated with a distinct train of symptoms, and *requiring distinct modes of treatment.*" 69. If this be the case, and if it be true, as our author thinks it is, "that both the *kind and the seat* of these morbid changes vary in the different cases," then we have a labyrinth to wade through which will require the stoutest heart to encounter, and which very few medical practitioners will attempt to unravel.

It is an extremely difficult matter to hit the happy medium between impracticable refinements and useless generalities in medical matters. With all our respect for the talents of Dr. Hall, we cannot but fear that he has verged occasionally to the former extreme. We do not indeed doubt that, with his own tact for minute observation, the distinctions which he has drawn, may be recognized, and even turned to useful account; but we question whether one practitioner in twenty will be able to follow him, or profit by him in some of his discriminations. The following quotation will shew that the *difference of treatment* between these different shades of disease really amounts to nothing.

"The remedies for these disordered states of the complexion are such as induce and continue alvine evacuations of ample quantity and natural colour. Mild mercurials are efficacious, in conjunction with mild purgative medicines: they require to be given for a very considerable length of time, and should not, therefore, be repeated too frequently.

"In other respects the treatment is similar to that of the other forms of disorder of the general health; the objects being to restore the healthy state of the bowels, and of the uterine discharges, to give vigour to the system, and, if necessary, to remedy the different local complications.

"Sponging, and much friction of the general surface, warm clothing, and a particular attention to guard against coldness of the feet, have appeared to me of great use in restoring the proper state and functions of the cutaneous surface." 74.

Thus we see that the same treatment is laid down for these three varieties, each of which was said to require a *dictinct mode of treatment*, while the methodus medendi in this trio really differs so little from that recommended in "the other forms of disorder," as to be scarcely cognizable.

### Chap. VI.—*Hysteria.*

This disease is divided by Dr. Hall into three forms or varieties—the mild, the severe, and the inveterate. The common mild form is so well known as to require no remark. The severe form, like Proteus himself, assumes such a variety of shapes and characters as to pass undetected in numerous instances, even under the eyes of the most experienced practitioners.

"The commencement, course, or termination of this, and indeed of every form, of this complaint, is generally marked, and the case distinguished, by the signs of some inordinate mental emotions, to which it is most important to revert, as they afford, in many cases, the most characteristic symptoms of this disorder.

"The attack is frequently ushered in by an unusual appearance of the countenance,—change of colour, rolling of the eyes, distortions, or some spasmodic affection of the muscles of the face.

"A state of general or partial, of violent, or of continued convulsion, or of fixed spasmodic contraction, takes place, and displays every possible variety in mode or form, as trismus, tetanus, contracted hand, distorted foot, or twisted legs.

"The severe form of hysteria sometimes consists chiefly in a violent general or partial pain and throbbing of the head; occasionally this pain is confined to some particular spot even, and is so acute as to have obtained the appellation of the clavus hystericus; sometimes there is great intolerance of light and noise; sometimes a state of stupor, sometimes delirium. This state must be carefully distinguished from an idiopathic affection of the head.

"The respiration is frequently much affected. An oppressive or suffocative dyspnœa takes place; or the breathing becomes rapid, anxious, and irregular, or variously attended with rapid heaving of the chest, or with a spasmodic affection of the diaphragm inducing a peculiar elevation of the abdomen, or an equally peculiar successory movement of the trunk in general; sometimes the respiration appears to be suspended for some time, the pulse continuing to beat as before; in this case it will generally be found, on attentive examination, that the breathing is performed by the diaphragm.

"A peculiar crowing noise or screaming is apt to occur in this affection; and there is occasionally great hoarseness, or even complete loss of voice, continued for a considerable time.

"There is sometimes a violent, dry, hoarse cough, continued or recurrent in paroxysms; and sometimes there is an attack which resembles the most suffocating form of croup.

"Palpitation of the heart, or syncope, are usual affections in hysteria; the pulse is, otherwise, often very little affected.

"There is occasionally acute pain of some part of the chest, of the diaphragm, or of the abdomen. This affection might easily be mistaken for inflammation. It is often such that the patient cannot bear the slightest, and most superficial touch even; and it is by this very circumstance that it is sometimes distinguished from pain of an inflammatory nature, which is only aggravated by positive pressure." 84.

The inveterate form of hysteria consists, according to Dr. Hall, sometimes in an almost perpetual agitation of some part of the body, the limbs, the respiration, the throat, or the stomach—sometimes in a state of perpetual contraction of the hand, foot, or some other part of the body. In some instances, there are seizures which it is difficult to distinguish from epilepsy, paralysis, or mental alienation. In short, "hysteria is characterised by affecting in the same, or in different instances, singly or conjointly, all the several systems which constitute the animal frame"—all the organs and functions of the body—all the faculties of the mind!

Yet it cannot be denied that it is of great importance to distinguish the masked forms of this malady from the inflammatory and organic diseases which it imitates. They are every day confounded, especially by young practitioners, and much mischief is done, besides the false prognoses that are given. Such errors are to be avoided by a cautious inquiry into the history of the case, the mode of attack, the immediate exciting cause, and the early symptoms. With all these precautions, however, the practitioner will often be deceived, and only discover his mistake in the course of the disease, when he finds that local affections, which he considered to be inflammatory, are aggravated instead of being relieved by depletion—especially sanguineous depletion.

The methodus medendi in hysteria laid down by our author forms a striking contrast to the minuteness of his descriptions.

"The remedies in hysteria are such as are required by the state of constitutional disorder, and especially by that of the stomach and bowels, and by that of the uterus, the functions of which require to be restored as quickly as possible. Aperient medicines, fomentations of the feet, and of the lower parts of the abdomen, are amongst the first remedies.

"Then follow the means of relieving the urgent symptoms, which are as various as those symptoms themselves.

"For the affection of the head, a lotion of spirit of wine and rose-water is of great service, and, if necessary, a small blister may be applied to the nape of the neck; with these remedies, the tincture of hyosciamus, sal volatile, and æther may be given, or a saline effervescent draught.

"For the pain of the chest or abdomen, a liniment with sal volatile or a fomentation of hot water, may be applied with similar internal remedies.

"The same observations apply to the other forms of hysteria. In all it is of the first importance to act upon the alimentary canal and uterus, then to soothe, and, lastly, to relieve the local pain or distress." 89.

In a short chapter (VII.) which succeeds, Dr. H. has offered some cursory observations on certain anomalous forms of disordered health in young females, all of which, however, appear to hinge upon defective energy in the stomach and bowels, and consequently are attended with debility, and more or less emaciation. Amenorrhœa is also a concomitant symptom. The remedies are mild aperients, tonics, slight cordials, with alternate repose and exercise.

Chap. VII. This embraces a disorder of general health which our author has observed in females returned from India.

Dr. Hall, arguing on the fact that menstruation occurs earlier in tropical than in cold climates, concludes that the uterus suffers in females who migrate from Europe to India. He observes that the catamenial flow is apt to become profuse in the hotter regions of the earth, attended, in many instances, with disposition to mænorrhagia, and even to abortion and leucorrhœa.

"The result of this state of things is, that the patient becomes affected with extreme exhaustion, and is often compelled to return to Europe. This change of climate is generally of the utmost service in restraining the uterine discharges; but the effects of the previous drain and losses of blood do not so soon cease, and the patient presents all the symptoms of exhaustion, either in that form which is attended with re-action, or in that in which the symptoms of re-action do not manifest themselves. In addition to exhaustion, there are also very frequently the effects of intestinal irritation, the bowels being extremely apt to become confined." 102.

Two cases in illustration are related by Dr. Hall, for which we refer to the work, from page 103 to 109. The treatment differs little, if at all, from that which has been already detailed.

Chap. VIII. This chapter, which only occupies nine pages, discusses "the diagnosis and symptoms of some local inflammatory diseases." Dr. H. commences by observing that it is the fashion of the present day to consider every local pain and other affection to be inflammatory, "and forthwith to use the lancet." Our author is anxious not to mislead any young practitioner by drawing him into a neglect of this useful remedy, when actual inflammation exists.

"I would even say that it is far better that the lancet should be used twenty times unnecessarily, than that it should be neglected once when really necessary. But still it is my duty to state, that I have seen many, very many cases of protracted indisposition which have entirely ensued from the misapplied and unnecessary use of the lancet." 112.

How are we to avoid the error? By a careful diagnosis.

"Whenever there are the appearances of the complexion, tongue, and general surface, which have been described as obtaining in the different forms of disorder of the general health, the presumption will be, that any local affection is only a symptomatic complication of that disorder. This presumption is strengthened if there be an entire absence of any external cause of the local affection. But it is to be carefully observed that it is, still, only a presumption, and that there may be a concurrence of idiopathic local disease with disorder of the general health,—or that that which was symptomatic at one period, may become actual disease in its course,—especially in the more acute form of disorder of the general health described in the second chapter of this work;—for this very rarely, perhaps never, occurs in the other forms of this disorder.

"If there be many of the general symptoms of disorder of the general health which affect the head, the heart, the breathing, the nervous and the muscular systems, &c. there is a still further, and, I think, a still stronger presumption that any predominant topical affection is symptomatic; for I have repeatedly observed that idiopathic disease frequently subdues these symptoms, even when they had previously existed, and gives a definitiveness to the affection which it had not before, and which returns only when the idiopathic disease is subdued." 114.

Dr. Hall would not say that hysteria is incompatible with idiopathic inflammatory disease; but he is persuaded that attacks or symptoms of hysteria very seldom concur with the active inflammation of a vital organ. It is sometimes superinduced by the remedies for inflammation, denoting then that those remedies have mitigated the disease and affected the constitution; but he believes that in the greater number of cases where hysteria comes on after bleeding, the original complaint was not inflammation, but disorder of the general health with some local complication.

"Another source of distinction exists in the effects of the remedies which have been employed. Early fainting, from blood-letting, is observed in the complications of disorder of the general health; inflammation, on the other hand, seems to protect the system from the effects of loss of blood: in the former case, too, there are often mitigated sufferings at first, but an aggravated state of complaint on the return of re-action after bleeding; whilst in the latter, this is certainly not observed, but the disease may be found to be unsubdued, or perhaps pursuing its progress." 116.

On the other hand, the complications of disorder of the general health are more relieved by purgatives than an inflammatory affection would be, while the appearance of the evacuations affords an additional source of diagnosis. The administration of lowering remedies in disorder of the general health renders the patient nervous, irritable, and feeble, in a degree not observed in inflammatory disease.

Dr. Hall makes many judicious observations on the diagnosis in particular local affections, as of the head, the pleura, the peritoneum, the lungs, &c. for which we must refer to the work itself.

CHAP. IX.—The subject of this chapter is tubercular affection of the abdomen. This is a most insidious, slow, and generally fatal disease. In Dr. Hall's experience, there are more females than males affected with the complaint. The age at which that peculiar form of the disease of which Dr. H. is now speaking, occurs, is usually, according to his experience, from fifteen. The following are the diagnostic symptoms by which our author is guided.

"Tuberculous disease in the abdomen is greatly characterized by three symptoms,—great tendency to coldness and lividity of the extreme parts of the body, a frequent pulse, and slow but progressive emaciation.

"The aspect of the countenance is altogether peculiar, especially in cool weather, together with an obvious emaciation and expression of languor and disease; the end of the nose is livid in colour, and cold to the touch; and there is, in general, either paleness or a slight degree of flushing.

"Similar observations may be made respecting the general surface. There is emaciation; the skin is soft, and apt to become moist, and there are frequently perspirations during sleep, especially in the early part of the morning; to prevent this perspiration, the patient frequently endeavours to keep awake; there is an undue sensibility to cold observed on the slightest unexpected exposure,—as the opening of a door,—and the patient frequently creeps over the fire; sometimes I have observed the back of the hands, and the fore part of the legs, to assume a peculiar brown colour, from being burnt by a constant approach to the fire; the hands and fingers are apt to be extremely livid and cold.

"The mode of walking is peculiar, being attended by stooping, weakness, and caution.

"The pulse is always frequent, and generally regular. It is earlier, and longer frequent, in tuberculous affection of the abdomen, than of any other cavity. I have known the pulse to be between one hundred and one hundred and twenty, for several years.

"The emaciation in tuberculous disease of the abdomen is uniformly but very slowly progressive. It is accompanied by a state of unvaried debility; and in the later periods of the disease, by some œdema, generally observed more in one leg than the other.

"The other symptoms of this morbid affection are less constant; they are chiefly an augmented appetite for food, copious, pale, alvine evacuations, and pain, and sometimes a perceptible tumor, in some part of the abdomen, especially in the iliac or hypogastric regions. The catamenia simply become scanty, or cease, without undergoing the changes observed in some cases of disorder of the general health.

"There are altogether a peculiar appearance of the countenance, a peculiar mode of walking, and a peculiar attitude and manner in general, all denoting debility and great disease; and if to them be added the peculiar sensibility to cold, and tendency to coldness and lividity of the extreme parts of the body, the very gradual emaciation, and the habitual frequency of the pulse, it is scarcely possible to mistake the nature of this disease; but in practice the diagnosis requires very careful and minute observation." 123.

As for tubercular affections of the encephalon they can only be suspected and distinguished from slow inflammation by observing the concurrent existence of tubercles in other parts of the body. The symptoms of tubercles in the lungs have been so ably described by Bayle, Laennec, and Louis, that our author does not think it necessary to take up the question here. Several cases are detailed by Dr. Hall, illustrating the graphic description of tuberculous affection of the abdominal cavity, which we have fully quoted. These cases do not require any analysis in this place.

Chap. X.—This chapter contains some observations on certain affections of the uterus and mamma. The uterus may be affected by disorder of the general health—by a state of exhaustion of the system—by organic disease of some important viscus—and by inflammation of the uterus itself. The morbid conditions which come in under the first head, consist in an impaired state of the catamenial function, supervening, for the most part, gradually—the uterine discharge becoming irregular, generally retarded in its returns, curtailed in its duration, defective in quantity and colour—often ending in complete amenorrhœa. To these symptoms, there is sometimes added painful menstruation, which is much relieved by pediluvia, semicupia, and warm water injections, with opium or hyosciamus.

But our author's chief object in this chapter is to notice some morbid states of the catamenial function, apparently dependent upon an inflammatory affection of the uterus itself, attended with amenorrhœa, dysmenorrhœa, and the ejection of a false membrane from the internal surface of the organ.

"All these cases are attended and denoted by pain. This is frequently constantly present, or at least easily excited by coughing, straining, or jumping; it is often felt on voiding the bladder or rectum: it is apt to be augmented in paroxysms, and it is sometimes attended by a sense of bearing down, by frequent calls to make water, or by some degree of tenesmus or uneasy feeling about the rectum; and it is not unfrequently attended by pain at the lowest part of the back, and round the pelvis.

"Amenorrhœa, arising from an inflammatory state of the uterus, will, if we can divest ourselves of the influence of a name, be readily distinguished from the cessation of the catamenia in chlorosis, by the absence of the peculiar appearances of this disorder, and by the pain and other symptoms of inflammation. The mode of accession of this case of amenorrhœa is different from that in chlorosis, being less gradual, and less marked by the gradual changes of colour in the discharge.

"Dysmenorrhœa from an inflammatory condition of the uterus is still more common, perhaps, than amenorrhœa. It is sometimes accompanied by great pain and profuse discharges, at each catamenial period, and often proves a cause of sterility. In one instance, the patient became pregnant at length; but the substance of the uterus was diseased, and presented the form of painful tumours, on examination of the abdomen; and, after delivery, a fatal inflammation destroyed the patient. On examination, the uterus was found to be the seat of a diseased structure, in a state of partial suppuration.

"In some instances, the inflamed lining of the uterus has formed a false membrane of coagulable lymph, such as is formed, in some cases, in the trachea, and in the intestines; and this is at length thrown off by painful contractions of the uterus, frequently accompanied or followed by hæmorrhagy. The case resembles abortion. The false membrane has the form of the internal cavity of the uterus, but is readily distinguished, on a careful examination, from an ovum.

"In all these cases, an active application of antiphlogistic remedies is absolutely necessary to relieve the patient, to subdue the inflammation, and to secure the organ from a future state of disease of a still more formidable character. I would particularly enforce this remark, in regard to all cases of dysmenorrhœa, which are too apt to be treated as a mere monthly inconveniency, without sufficient reference to the nature and tendency of the complaint; we ought, on the contrary, to be satisfied with nothing short of totally subduing the disease.

"For the case of inflammation, in which a false membrane is apt to form, judging from analogy, I should imagine that a full course of mercury might cure. I am not aware that it has been tried. But considering the painful character of the affection, and its usual effect in inducing sterility, it appears to me that this remedy deserves to be submitted to the test of experiment.

"Otherwise, I am not aware of any mode of treatment, except that adapted for inflammation and pain, in general; only, I would suggest, that the antiphlogistic measures should not be adopted during the attack merely, but should be continued long afterwards; for the formation of the false membrane is the more immediate effect of inflammation, whilst its detachment and expulsion are more properly the consequences of contraction of the uterus itself." 140.

We have thus given a very full analysis of the first part of our author's work, embracing "the Disorders incident to Female Youth." In a succeeding article, we shall take up the important subject of puerperal diseases. In the mean time, we have given sufficient specimens of the minute and valuable observations of this talented author, to induce the practitioner to possess himself of the original work, which is, in this edition, put into a much more systematic and available form than in Dr. Hall's preceding volumes.

